# Bidirectional Piezoelectric Energy Harvester

**DOI:** 10.3390/s19183845

**Published:** 2019-09-06

**Authors:** Andrius Čeponis, Dalius Mažeika, Artūras Kilikevičius

**Affiliations:** 1 Department of Engineering Graphics, Faculty of Fundament Sciences, Vilnius Gediminas Technical University, Sauletekio avn., 11, LT-10223 Vilnius, Lithuania; 2 Department of Information Systems, Faculty of Fundament Sciences, Vilnius Gediminas Technical University, Sauletekio avn., 11, LT-10223 Vilnius, Lithuania; 3 Institute of Mechanical Science, Vilnius Gediminas Technical University, J. Basanaviciaus str. 28, LT-03224 Vilnius, Lithuania

**Keywords:** energy harvesting, bidirectional excitation, cantilever array

## Abstract

This paper represents a numerical and experimental investigation of the bidirectional piezoelectric energy harvester. The harvester can harvest energy from the vibrating base in two perpendicular directions. The introduced harvester consists of two cantilevers that are connected by a particular angle and two seismic masses. The first mass is placed at a free end of the harvester while the second mass is fixed at the joining point of the cantilevers. The piezoelectric energy harvester employs the first and the second out of plane bending modes. The numerical investigation was carried out to obtain optimal geometrical parameters and to calculate the mechanical and electrical characteristics of the harvester. The energy harvester can provide stable output power during harmonic and impact-based excitation in two directions. The results of the investigations showed that energy harvester provides a maximum output power of 16.85 µW and 15.9 4 µW when the base has harmonic vibrations in *y* and *z* directions, respectively. Maximum output of 4.059 nW/N and 3.1 nW/N in *y* and *z* directions were obtained in case of impact based excitation

## 1. Introduction

Nowadays, wireless electronic devices, networks of wireless sensors, biomedical devices, and wearable electronics take a huge part in most areas of industry, life sciences, medicine, etc. [1,2]. However, wireless and portable devices have the main disadvantage—short battery life, and as a result, it leads to environment pollution, high costs of maintenance and etc. Fortunately, nowadays, conventional batteries can be replaced partly or fully by kinetic energy harvesters [3,4]. There exist four main kinetic energy harvesting mechanism: electromagnetic, electrostatic, triboelectric, and piezoelectric. Each kinetic energy transduction mechanism has disadvantages like electromagnetic kinetic energy harvesters have low-level output voltage, are not suitable for high excitation frequencies and are not suitable for MEMS applications. Electrostatic energy harvesters have demand on external power supply, extremely high isolation ratio, and have a complex structure. Triboelectric energy harvesters provide low-level electrical characteristics which are, in most cases, not suitable for direct delivery to load [5,6,7,8]. On the other hand, energy harvesters based on piezoelectric materials have numerous advantages like high output power densities, piezoelectric energy harvesters are suitable for MEMS applications, in most cases, harvesters are magnetic field free and have a simple structure and etc. [9,10]. Therefore, due to these advantages, piezoelectric energy harvesters attracted a lot of attention during the last decade. Usually, investigations of piezoelectric energy harvesters are focused on the improvement of mechanical and electric characteristics of piezoelectric cantilevers. Disadvantages like narrow bandwidth and uneven strain distribution at piezoelectric material have been investigated extensively. However, can be noticed, that in most cases, the excitation force vector is perpendicular to the harvesters mounting plane [11,12,13]. In real-life applications, energy harvester designed to operate with ideally aligned excitation force can face operation issues due to dynamic changes in the direction of excitation force. Therefore, investigations on piezoelectric energy harvesters which operation is based on bidirectional excitation force become highly relevant to obtain possibility employ energy harvesters at real environments with the varying direction of excitation.

Fan and et al. reported on multipurpose piezoelectric energy harvester. The harvester is based on beam-roller structure which is able to operate with sway and bidirectional excitation forces. Proposed harvester has a simple structure which is based on the cylinder, piezoelectric cantilever with magnetic coupling and clamping frame. Operation of the harvester is based on the first bending mode of the cantilever. Authors claimed that such structure can harvest kinetic energy from two perpendicular excitation forces at the same time. Also, the proposed structure exhibits frequency up functionality i.e., converts low-frequency sway vibrations to higher frequency vibrations suitable to drive piezoelectric cantilever [14].

Mei and Li reported on cylindrical double-wall multi-modal piezoelectric energy harvester for multi-directional energy harvesting. The design of harvester is based on two steel hollowed cylinders placed inside each other and seismic mass. The active material is located on the outer surface of the inner cylinder. Operation of the harvester is based on eight vibration modes. Therefore, multidirectional operation of the energy harvester is obtained by the employment of different vibrations modes which have differed deformation directions. However, the design of cantilever has the main disadvantage, i.e., low strain amplitudes and its distribution across active material due to the high stiffness of cylinders [15].

Yang and et al. investigated frame based two-directional piezoelectric energy harvester. The energy harvester is based on three cantilevers connected to U shaped frame and seismic mass placed in the center of the horizontal cantilever. Operation of the energy harvesting structure is based on two fundamental vibration modes, i.e., vertical and horizontal. On basis of numerical and experimental investigations, the authors concluded that that proposed energy harvester could capture vibration energy from arbitrary directions in a 2D plane. Also, the authors claimed that if structural parameters of the harvester are well adjusted, the system can provide a suitable power output level for practical applications [16].

Wang and et al. introduced a two-directional energy harvester with the ability to harvest kinetic energy in two in-plane directions using a flexible cylinder with radially distributed piezoelectric patches. The harvester has simple design i.e., fixed end cylinder made of polyurethane, four piezoelectric patches which are placed near the fixed end of the cylinder. Based on numerical and experimental investigations, the authors concluded that the proposed design of energy harvester could harvest vibration energy in the arbitrary in-plane direction and provide stable power output irrespective to angle of excitation force vector. Also, the authors introduced a new approach named angle bandwidth. This approach gives the possibility to describe harvester’s ability to harvest bidirectional vibration energy [17].

Karami and Inman reported on modeling and experimental investigations of the zig-zag shaped energy harvester. The harvester contains a collection of cantilevers, with rectangular cross-sections, placed next to each other on the main plane. Each cantilever is connected to the neighboring cantilever and can have out-of-plane vibrations and to twist. Based on the investigations it can be assumed that energy harvester can capture bidirectional vibrations of two vibration modes. However, the authors did not investigate harvester’s ability to harvest bidirectional vibrations [18,19].

Abdelmoula and et.al. investigated zig-zag shaped piezoelectric energy harvester as well. The design of harvester is similar to introduced by Karami and Inman [18,19]. The authors investigated harvesters ability to harvest the energy of torsion deformations and compered obtained results with bending mode. In conclusion, the authors stated that the torsion deformation mode is more effective compared to the bending mode. Based on numerical and experimental investigations it can be assumed that proposed energy harvester can be used to harvest bidirectional vibrations by torsional and bending modes. However, the authors do not mention an intention to investigate bidirectional operation principle [20].

This paper introduces numerical and experimental investigations on bidirectional energy harvester. To obtain suitable geometrical parameters of the harvester, the multiparametric problem with six geometrical variables has been solved. Further, the electrical and mechanical characteristics of the harvester have been investigated in detail. Results of the investigations revealed that the proposed design of the energy harvester can provide stable power output during bidirectional harmonic and impact based excitations.

## 2. Design and Operation of Bidirectional Energy Harvester

**Figure 1:**
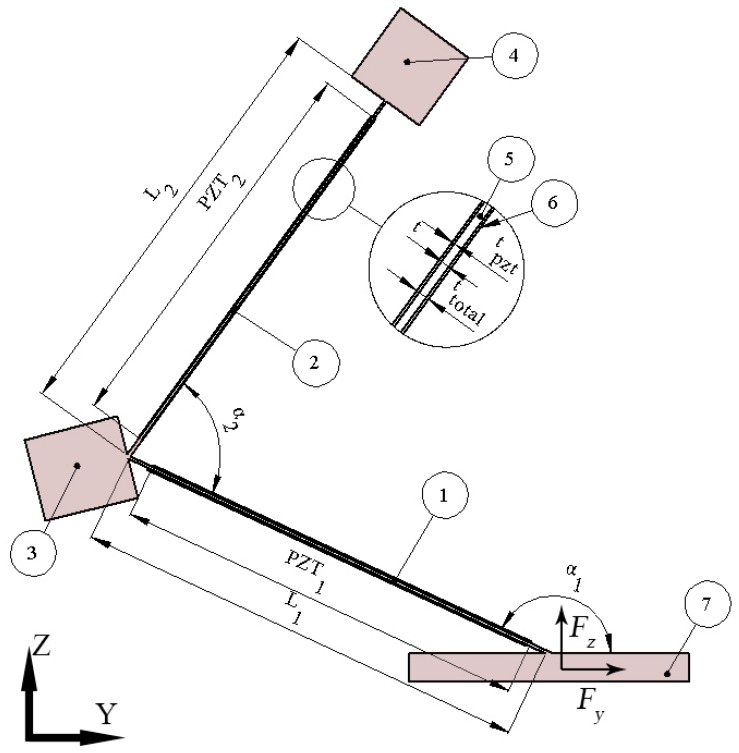
Schematics of bidirectional energy harvester; 1,2—the first and second cantilever, respectively; 3,4—seismic masses, M_1_ and M_2_, respectively; 5—body of the harvester; 6—piezoelectric material made of PIC255 piezo ceramics; 7—clamping base.

The proposed energy harvester consists of two bimorph piezoelectric cantilevers that are connected by angle α_2_ (Figure 1(1,2)). The lower cantilever is rigidly clamped to the base under angle α_1_. Specific angles give possibility to increase rotation moment when bidirectional vibrations are used. Two seismic masses are included in order to increase rotation moment and to decrease resonant frequencies of the harvester (Figure 1(3,4)). The first seismic mass M_1_ is placed at the junction between two cantilevers while the second seismic mass M_2_ is fixed at the free end of the structure.

All geometrical and physical parameters of the piezoelectric energy harvester are listed in Table 1 and Table 2. The geometrical parameters were obtained by solving multiparametric optimization problem with six design variables. The optimization problem is described in Section 3. The initial values of the harvester used for optimization are listed in Table 3.

Operation of the energy harvester is based on the first and second out of plane bending modes of the structure. The energy harvesters are able to harvest energy when external force is applied to the base in *z* or *y* directions. The harvester operates as a spring with two masses attached by lever arms when vibration force is applied in *z* direction. Misbalance of the system is created by seismic masses, specific angles α_1_ and α_2_. Such design of the harvester allows to increase rotation moment which leads to improved electrical characteristics by increased strain distribution.

When external force is acting in *y* direction then energy harvester can be described as the spring with additional seismic massed attached to it by lever arms and effected by force perpendicular to axis of the spring. In both excitation cases the first and second out of plane bending modes are obtained and rotation moments are increased because of seismic masses and specific angles α_1_ and α_2_ that increase bending strain at piezo ceramic layers.

Also, it must be mentioned that cantilever L_2_ can operate as a dynamic vibration damper (DVA) for cantilever L_1_. Numerous reports of DVA applications in piezoelectric energy harvesters can be found [20,21,22]. Operation of cantilever L_2_ as DVA is avoidable for the proposed energy harvester, therefore, it was controlled that proper geometrical parameters of cantilevers would not be selected during the numerical investigation.

## 3. Results of Numerical Investigation

Numerical investigation of bidirectional energy harvester was performed in order to solve the following tasks: To obtain optimal geometrical parameters of the harvester by solving optimization problem, indicate the highest possible excitation acceleration amplitude, verify operation principle of the harvester and to calculate mechanical and electrical output characteristics. For this purpose, finite elements model (FEM) was built by employing Comsol 5.4 software. Structure of the harvester was made from aluminum 6061 alloy while PIC225 soft piezoelectric ceramic (Physik Instrumente GmbH, Germany) was used for piezo ceramic elements. Material properties are presented in Table 1.

**Figure 2:**
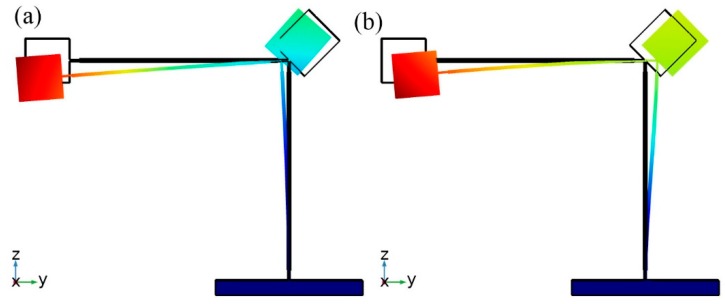
Vibration modes of the harvester with initial parameters; (**a**)—modal shape at 29.858Hz; (**b**)—modal shape at 41.257Hz.

Initial values of the parameters used for the optimization of the bidirectional energy harvester were set up (Table 2). Modal analysis of the harvester was performed to find modal shapes and natural frequencies when initial values are used. The results are shown in Figure 2.

The first and second bending modes are the most suitable for bidirectional operation, therefore, optimization of the harvester will be performed with aim employ these modes for bidirectional energy harvesting.

Optimization problem has been divided into three steps. These steps were performed sequentially, applying frequency-domain study when the acceleration of the base was aligned to the *z* and *y*-axis. The excitation frequency range was analyzed from 10 Hz to 100 Hz. Acceleration amplitude of the base was set to 0.1 m/s^2^.

The length of cantilevers *L*_1_ and *L*_2_ were optimized in the first step. Seismic masses (*M*_1_ and *M*_2_) and angles (*α*_1_ and *α*_2_) were set to initial values which are given in Table 2. Maximization of average von Mises stress along both cantilevers was chosen as an objective of the optimization problem:

(1) $$\underset{l}{\max}\left(\sigma_{avg}\left(l\right)\right)$$

subjected to

(2) $$L_{\min}\leq L_{i}\leq L_{\max}$$

(3) $$\sigma_{avg}<\sigma_{t}$$

where

(4) $$l=\left(L_{1},L_{2}\right)$$

here *σ_avg_* average stress inducted along the cantilevers; *σ_t_* tensile strength of 6061 aluminum alloy; *L*_1_ and *L*_2_ are length of the cantilevers; *L*_min_ and *L*_max_ defines minimum and maximum length values of the cantilevers. *L*_min_ was set to 75 mm while *L*_max_ was set to 85 mm.

**Figure 3:**
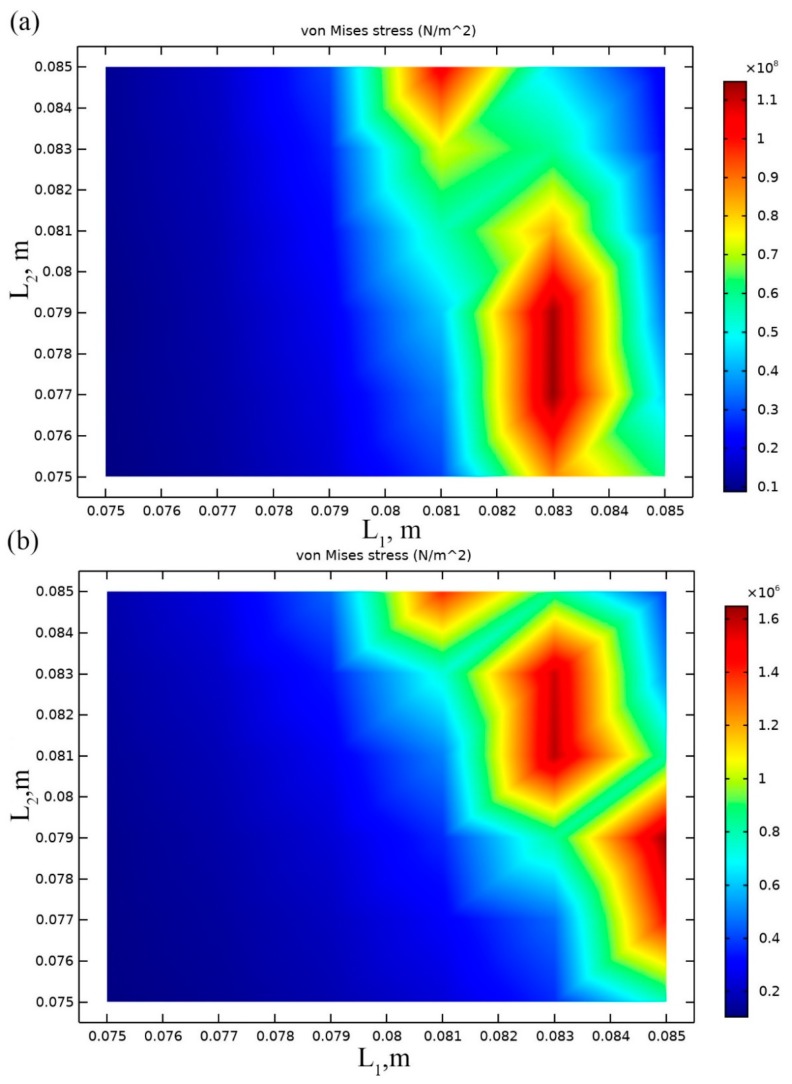
Plot of von Mises stresses at the resonance frequency when different *L*_1_ and *L*_2_ values are used: acceleration aligned to *z* direction, resonant frequency 24 Hz (**a**); acceleration aligned to *y* direction, resonant frequency 33 Hz (**b**).

Results of calculations are presented in Figure 3a,b where von Mises stress are shown at resonant frequencies when different values of *L*_1_ and *L*_2_ and base acceleration direction align to *z* and *y* axis.

Analyzing results shown in Figure 3a it can be noted that the highest average von Mises stress value of 1.2 × 10^8^ N/m^2^ has been obtained when cantilever lengths are *L*_1_ = 83 mm and 77 mm < *L*_2_ < 79 mm. It means that *L*_1_ must be kept at a particular value, while *L_2_* can be selected within the defined range. It gives flexibility in the combination of cantilevers lengths while excitation directions and resonant frequencies are different.

Figure 3b shows that the highest average von Mises stress value of 1.65 × 10^6^ N/m^2^ is obtained when *L*_1_ = 83 mm, while 80 mm < *L*_2_ < 83 mm. It can be noticed that the maximum average von Mises stress value is notably lower compared to the case with excitation in *z*-axis. Such difference occurred because of the *α*_1_ and *α*_2_ angles and different bending forces, respectively. As the length value of cantilever *L*_1_ is the same in both excitation cases, it was used as optimal. However, the length value of cantilever *L*_2_ does not match when different excitation direction is applied. As optimal *L*_2_ value was selected length value of 79 mm, considering that average von Mises stress values are notably higher compare when acceleration in *z* direction applied. Also, this value is the closest possible value to the range of *L*_2_ values when acceleration direction coincides with *y* direction.

Next optimization step was arranged to find optimal masses of seismic masses placed at tip and at the junction between cantilevers. Length of the cantilevers were set to optimal values obtained during previous investigation i.e., *L*_1_ = 83 mm; *L*_2_ = 79 mm while angles (*α*_1_ and *α*_2_) were set to initial values which are in Table 2. Average von Misses stress along the both cantilevers was chosen as objective of the optimization problem as in the previous step. The optimization problem can be written as follows:

(5) $$\underset{m}{\max}\left(\sigma_{avg}\left(M\right)\right)$$

subjected to

(6) $$M_{\min}\leq L_{i}\leq M_{\max}$$

(7) $$\sigma_{avg}<\sigma_{t}$$

where

(8) $$m=\left(M_{1},M_{2}\right)$$

here *σ_avg_* average von Misses stress inducted along cantilevers; *σ_t_* tensile strength of 6061 aluminum alloy; *M*_1_ and *M*_2_ are masses of the seismic masses; *M*_min_ and *M*_max_ defines minimum and maximum values of seismic masses mass. Lower bound of the interval *M*_min_ was set to 3 g and *M*_max_ was set to 6 g.

**Figure 4:**
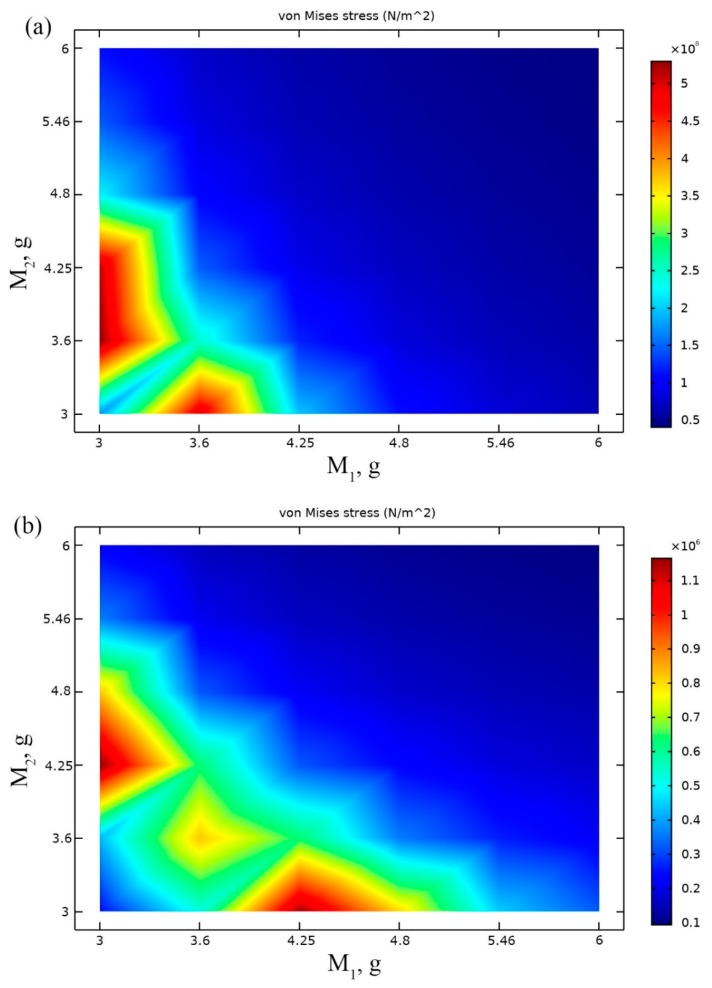
Plot of von Mises stresses at the resonance frequency when different *M*_1_ and *M*_2_ are used: acceleration aligned to *z* direction, resonant frequency 15 Hz (**a**); acceleration aligned to *y* direction, resonant frequency 28 Hz (**b**).

Results of calculations are presented in Figure 4a,b where von Mises stress are shown at resonant frequencies when different masses values and base acceleration direction align to *z* and *y* axis.

Analysis of Figure 4a showed that the highest average von Mises stress value of 5.5 × 10^8^ N/m^2^ was obtained at the resonant frequency of 15 Hz. This value has been obtained when *M*_1_ = 3.04 g and 3.3 g < *M*_2_ < 4.25 g. It can be noted that von Mises stress value is more than 5 times higher compared to the result obtained in the previous step of optimization. Moreover, resonant frequencies of the harvester slightly decreased.

During analysis of the results presented in 4b, it can be noted that maximum average von Mises stress value is 1.1 × 10^6^ N/m^2^ while excitation frequency was set to 28 Hz. This stress value is slightly lower compared to the result obtained in the *L*_1_, *L*_2_ optimization step. Two optimal pair of seismic mass *M*_1_ and *M*_2_ values was obtain i.e., *M*_1_ = 3.04 g, *M*_2_ = 4.25 g and *M*_1_ = 4.25 g, *M*_2_ = 3.04 g. It can be concluded that values of optimal seismic masses for excitation in *z* and *y* directions are in coincidence and can be used for both directions. We decided to select the first pair of mass values *M*_1_ = 3.04 g, *M*_2_ = 4.25 g for further investigation.

The last step of optimization was performed to find optimal angles *α*_1_ and *α*_2_. Length of the cantilevers and mass of seismic masses were set to optimal values obtained during previous investigations i.e., *L*_1_ = 83 mm; *L*_2_ = 79 mm and *M*_1_ = 3.04 g, *M*_2_ = 4.25 g, respectively. Average von Misses stress along the both cantilevers was chosen as objective of the optimization problem as in the previous steps. Optimization problem can be written as follows:

(9) $$\underset{m}{\max}\left(\sigma_{avg}\left(A\right)\right)$$

subjected to

(10) $$\alpha_{\min}\leq \alpha_{i}\leq \alpha_{\max}$$

(11) $$\sigma_{avg}<\sigma_{t}$$

where

(12) $$A=\left(\alpha_{1},\alpha_{2}\right)$$

here *σ_avg_* average von Mises stress inducted along cantilevers; *σ_t_* tensile strength of 6061 aluminum alloy; *α*_1_ and *α*_2_ are angles; *α*_min_ and *α*_max_ defines minimum and maximum values for the angles. Minimum value *α*_min_ was set to 1.13 rad and maximum value α_max_ was set to 2.7 rad.

**Figure 5:**
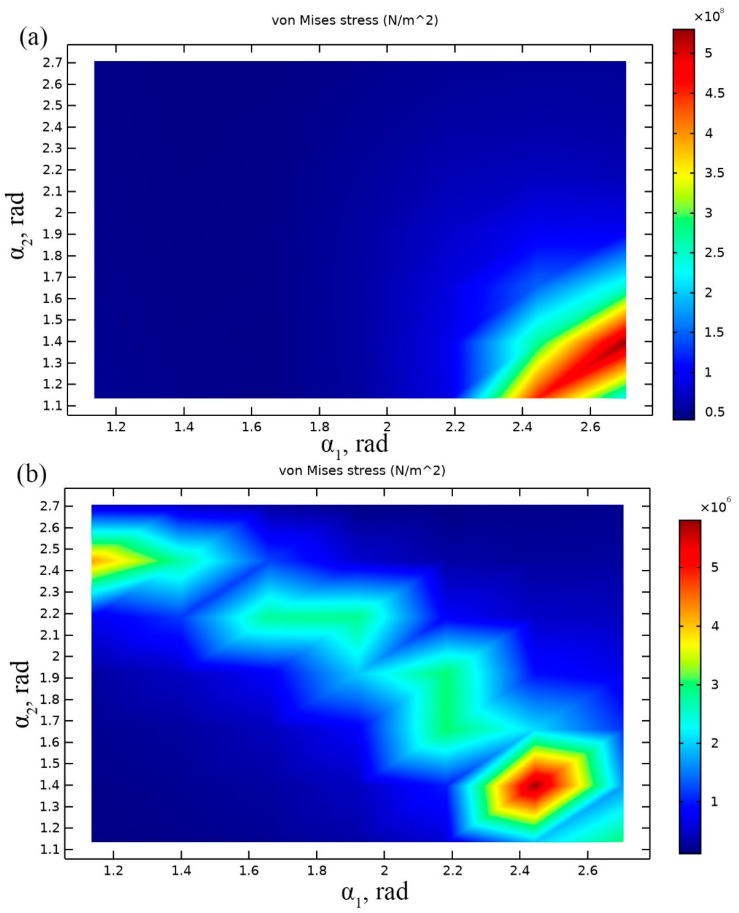
Plot of von Mises stresses at the resonance frequency when different *α_1_* and *α_2_* are used: acceleration aligned to *z* direction, resonant frequency 14 Hz (**a**); acceleration aligned to *y* direction, resonant frequency 27 Hz (**b**).

Results of calculations are presented in Figure 5a,b where von Mises stresses are shown at resonant frequencies when different angle values and base acceleration direction align to *z* and *y* axis.

Analysis of Figure 5a showed that optimal values of angles *α*_1_ and *α*_2_ are 2.75 rad and 1.38 rad, respectively. Average von Mises stress of 5.8 × 10^8^ N/m^2^ is obtained in this case. Optimal angles allowed improve of average von Mises stress value by 0.3 × 10^8^ N/m^2^ or 5.17%.

When the direction of the base acceleration was aligned to the direction of *y* axis, the largest average von Mises stress of 6.8 × 10^6^ N/m^2^ was obtained when *α*_1_ = 2.45 rad and α_2_ = 1.38 rad. The average stress value has increased about 5 times compared to the previous results. It shows that these angles ensure notable stress increment. However, obtained *α*_1_ values do not match when acceleration is applied in *z* and *y* directions. We decided to select α_1_ value of 2.75 rad because higher average von Mises stress value was obtained when acceleration direction coincide with *z* axis.

The optimal geometrical parameters of the bidirectional energy harvester was obtained and are listed in Table 3.

**Figure 6:**
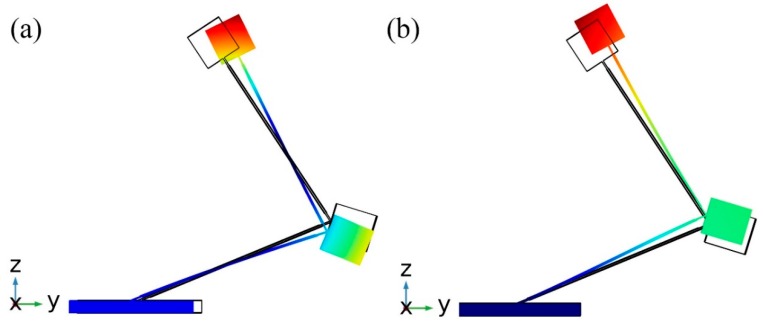
Vibration modes of bidirectional energy harvester; (**a**)—vibration shape at 13.825 Hz; (**b**)—vibration shape at 25.8 7Hz.

Therefore, in order to verify operation principle and clarify vibration modes, modal analysis of the optimized energy harvester was performed. Results of the modal analysis are given in Figure 6. It can be seen that energy harvester operates at the first and second vibration mode. During excitation in *y* direction vibration mode at 13.825 Hz will be dominant and will generate the most power output. On the other hand, during excitation in *z* direction vibration mode at 25.87 Hz becomes dominant and also will generate the power output. Domination of the vibration modes depends on excitation direction and it is related with the shifted mass center and additional rotation moment created by these masses. The shift of the mass center occurs because of the angles between cantilevers, while rotation is obtained by different values of seismic masses. Moreover, rotation moment magnifies bending and increase stress inducted in the cantilevers. Therefore, the first vibration mode is dominant during excitation in *y* direction due to the bending and rotation of the harvester. The second vibration mode is dominant only due to bending while rotation moment much lower when excitation in *z* direction is applied.

**Figure 7:**
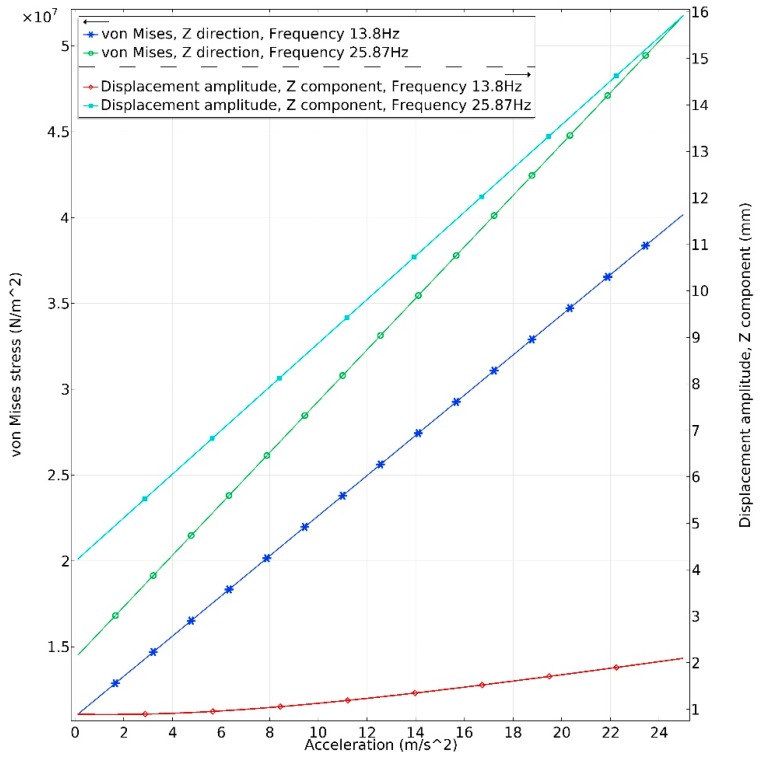
Dependence of maximum von Mises stress and tip displacement from acceleration in *z* direction.

**Figure 8:**
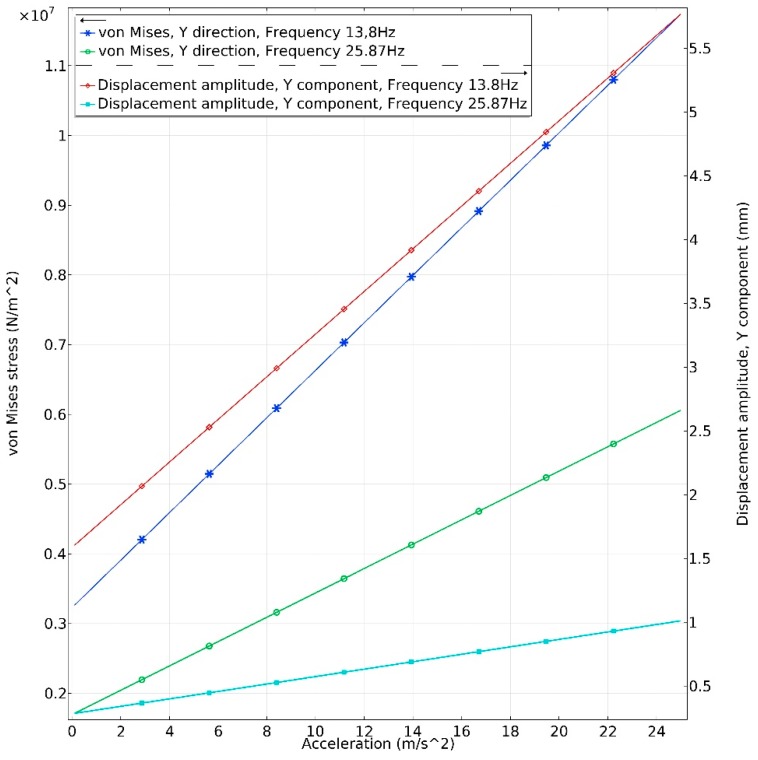
Dependence of maximum von Mises stress and tip displacement from acceleration in *y* direction.

In order to analyze reliability of the proposed energy harvester, dependence of the von Mises stress of the harvester from acceleration amplitudes in *y* and *z* directions was investigated (Figure 7 and Figure 8). Acceleration range was set from 0.1 m/s^2^ to 25 m/s^2^. Step size was 0.1 m/s^2^. The investigation was performed at two resonant frequencies i.e., 13.8 Hz and 25.87 Hz. It was considered that PZT-5 piezoceramic tensile stress is 50∙MPa, and it was examined that obtained stress does not exceed tensile stress when acceleration is increasing.

Analyzing results presented in Figure 7, it can be noted that the upper limit of acceleration in *z* direction is 23 m/s^2^ when host excitation frequency is 13.8 Hz. The maximum stress of 42∙MPa is obtained while the displacement of the tip is 1.8 mm. The upper acceleration limit of the harvester is 22.3 m/s^2^ at the host excitation frequency of 25.8 Hz. The maximum stress of 48∙MPa is obtained while tip displacement is 14.8 mm.

Figure 8 shows the maximum von Mises stress inducted in energy harvester during excitation in the *y* direction at resonance frequencies of 13.8 Hz and 25.8 Hz. The maximum stress of 12.5∙MPa and tip displacement of 5.8 mm is obtained at the frequency of 13.8 Hz when acceleration amplitude was 25 m/s^2^. The maximum stress of 0.6∙MPa and tip displacement of 1.1 mm was obtained at the resonance frequency of 25.8 Hz when acceleration amplitude was 25 m/s^2^.

Based on the obtained results, it can be concluded that energy harvester can operate at high acceleration amplitude. However, it must be noted that numerical simulation was performed, considering that vibrations are linear. Large tip displacement values obtained when excitation in the *z* direction is applied at a frequency of 25.8 Hz indicate that fatigue problem can occur. Therefore, the maximum acceleration value must be less than mentioned before. Although the proposed energy harvester is suitable for high acceleration values, we focused on the low-level excitation amplitudes. Therefore, the main results presented in this paper were obtained when acceleration was 0.1 m/s^2^.

To prevent the proposed energy harvester from possible failure at high acceleration amplitude, electronic damping can be used. Additional resistive, capacitive or inductive shunt circuit can be connected in parallel to one of piezoceramic plate in order to damp vibrations of the harvester and dissipate electrical energy as heat. Also, active damping systems can be used for the same purpose, i.e., an out-of-phase actuation can be generated applying external DC or out-of-phase AC voltage to one of the piezo ceramic plates to prevent the harvester from failure.

**Figure 9:**
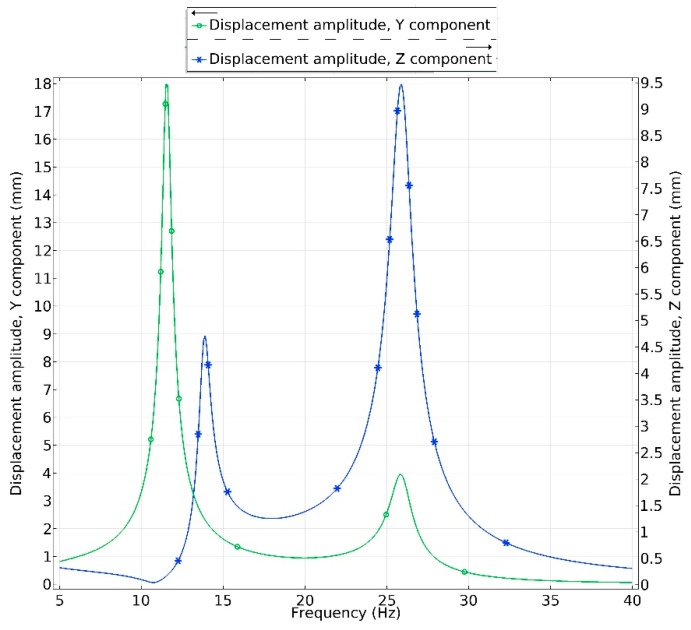
Displacement amplitude—frequency characteristics of bidirectional energy harvester.

Amplitude-frequency characteristic of the energy harvester was calculated when acceleration in *y* and *z* direction was applied. Frequency range from 5 Hz to 40 Hz was analyzed when acceleration was set to 0.1 m/s^2^. Results are shown in Figure 9.

Displacement amplitude—frequency characteristics was calculated for point located at free end of the energy harvester. Calculated resonant frequencies are in good agreement with the results obtained during modal analysis. It can be found that during excitation in *y* direction the highest displacement amplitude, 17.7 mm, was obtained at the first resonance. It shows that energy harvester will be able deliver the highest electrical characteristics during excitation by force with 13.825 Hz frequency and vector aligned to the *y* axis. On the other hand, the second resonance also will be employed. However, according to the displacement amplitude value equal to 4.1 mm, the electrical characteristics will be much lower compare to the first resonance. Therefore, it can be concluded that during excitation in *y* direction the best characteristics will be obtained during harvesters operation at the first resonance.

During harvester’s excitation in *z* direction, the highest displacement amplitude was obtained at 25.87 Hz frequency i.e., the second resonance. Displacement amplitude at this frequency reached 9.3 mm. As in the case before can be assumed that the highest electrical characteristics will be obtained at this frequency. Also, displacement amplitude—frequency characteristic shows that the first resonance is also employed. However, displacement amplitude is much lower i.e., 4.9 mm.

The results show that bidirectional energy harvester will be able to deliver energy during excitation in *y* and *z* directions by employing both resonances. However, it can be assumed that electrical characteristics will be unstable considering to excitation direction, vibration mode, excitation frequency and different stiffness of structure in *y* and *z* directions.

**Figure 10:**
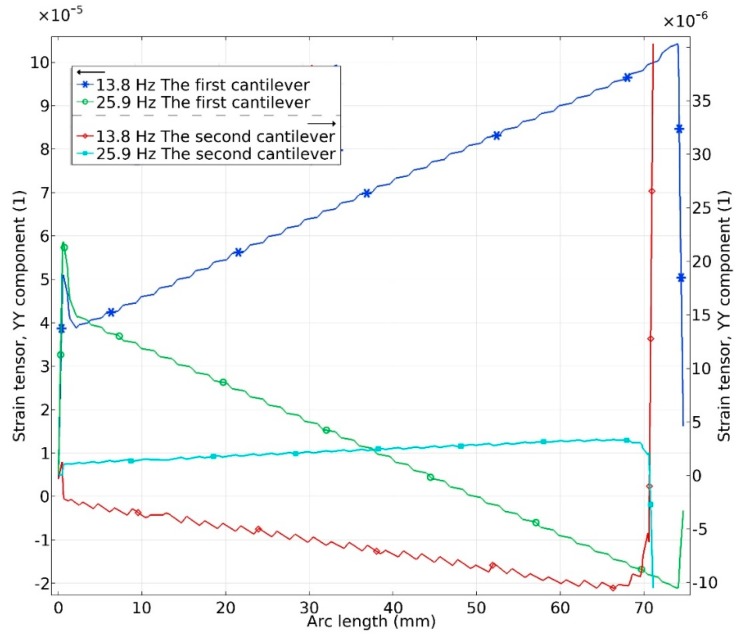
Bending strain characteristics at resonant frequencies during base excitation in *y* direction.

**Figure 11:**
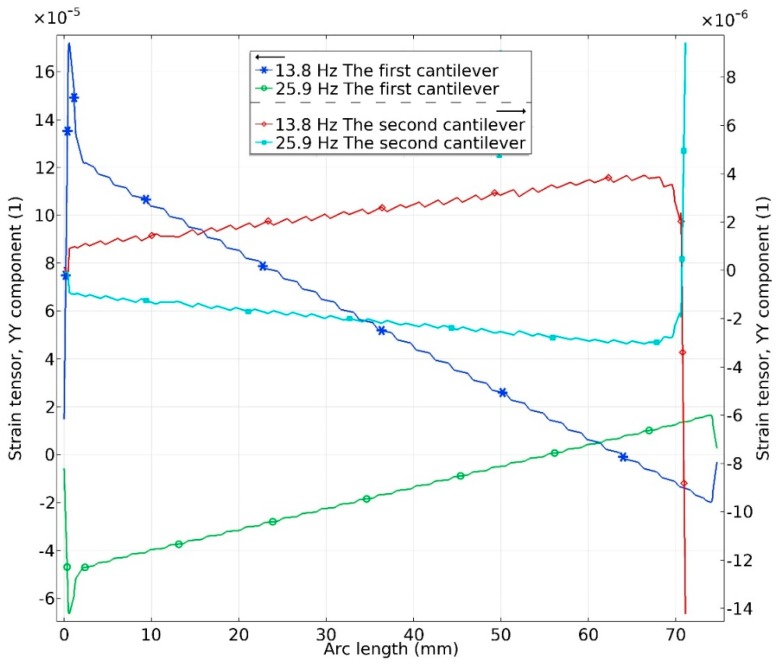
Bending strain characteristics at resonant frequencies during excitation in *z* direction.

In order to accurately assess mechanical characteristics of the harvester, bending strains inducted in piezo ceramic were investigated. An investigation was performed on each cantilever separately during base excitation in *y* and *z* directions. Results are given in Figure 10 and Figure 11.

Analysis of graphs presented in Figure 10 showed that considering to the resonant frequency, differences in bending strain values between the first and second cantilevers are around 10 times. The bending strain characteristics shows that the most bending strain is concentrated at the first cantilever. It can be assumed that the first cantilever will generate the most of electrical power while the second cantilever will generate much lower output ant will be more passive. However, it acts as an additional mass attached to the first cantilever thru lever arm and ensures additional rotation moment an as a result the higher electrical characteristics of whole energy harvester.

As can be found in Figure 11 the highest bending strain values are obtained at first cantilever. As in the case with excitation in *y* direction, differences in bending strain values between the first and second cantilevers are more than 10 times. According to Figure 11 it can be concluded that the highest bending strain values are at the first cantilever while the strain inducted in the second cantilever is notably lower. As in the case with excitation in the *y* direction, most of the electrical energy will be generated by the first cantilever while the second cantilever will provide much lower electrical characteristics. In summary of this part of investigations, can be concluded that the most of power will be generated by the first cantilever during excitation in *y* and *z* directions, while the second cantilever acts as an active system which ensures capture of excitation forces with different directions as well as delivers additional power output.

**Figure 12:**
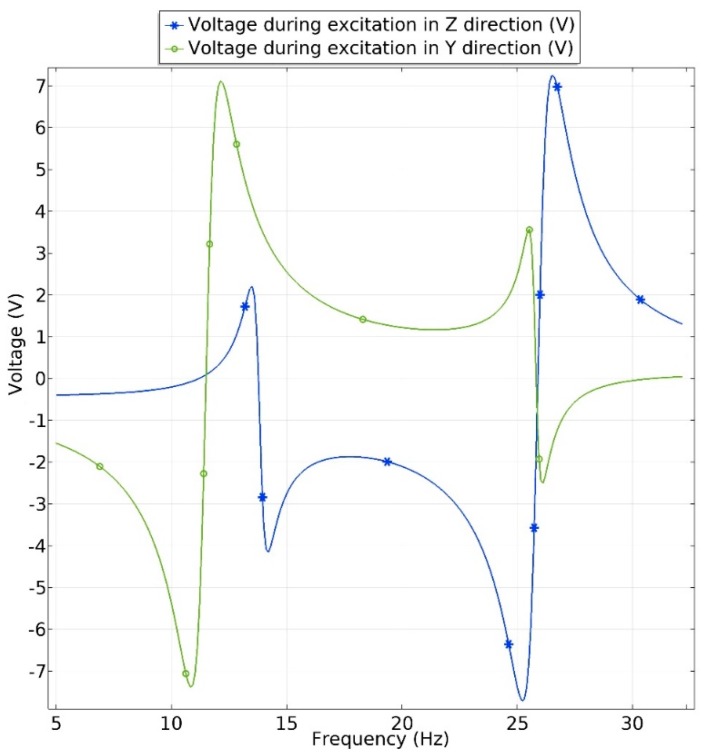
Total output voltage of bidirectional energy harvester.

The output voltage of the energy harvester was investigated at the frequency range from 5 Hz to 35 Hz. Acceleration was set to 0.1 m/s^2^. Results of the calculations are given in Figure 12. It can be seen that output voltage during excitation in *y* direction has the highest value at the first resonant frequency. It value reached 7.3 V_p-p_, while voltage of 2.8 V_p-p_. is obtained at the second resonant frequency. During excitation in *z* direction, the highest output voltage was obtained at the second resonant frequency. It value reached 7.1 V_p-p_, while output voltage at the first resonant frequency was 3.1 V_p-p_. Obtained results of output voltage confirm results given in Figure 10 and Figure 11 and correspond to bending strain distribution. Moreover, obtained results shows that energy harvester is able to provide a stable output voltage at different resonant frequencies. Also, it shows that energy harvester is able to operate with excitation forces aligned to the different axis and provide stable electrical outputs.

## 4. Experimental Investigation

**Figure 13:**
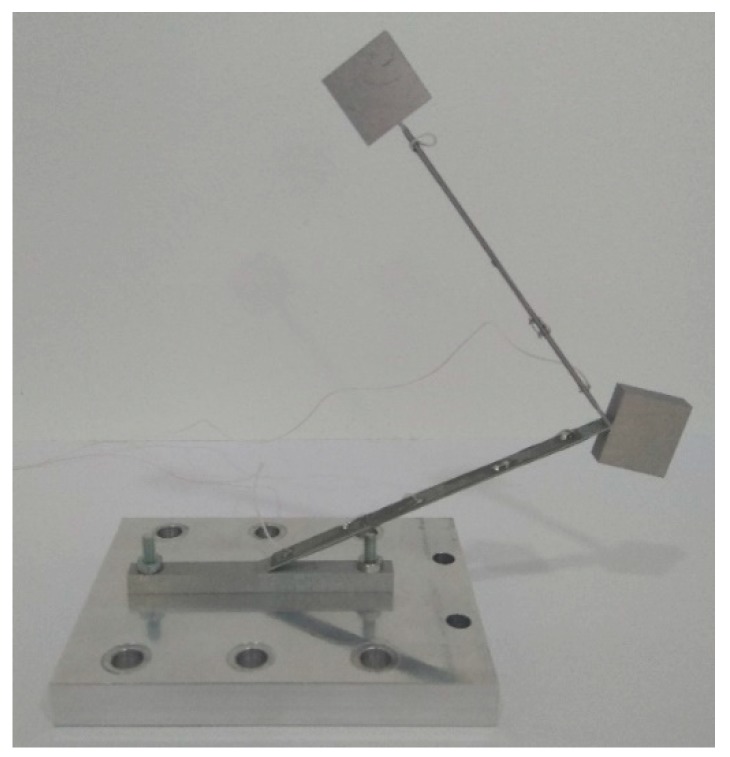
Prototype of bidirectional piezoelectric energy harvester.

An experimental investigation was performed to measure the electrical and mechanical characteristics of bidirectional energy harvester. A prototype of the harvester was made (Figure 13). Geometrical parameters and material properties of the prototype correspond to the characteristics given in Table 2 and Table 3.

**Figure 14:**
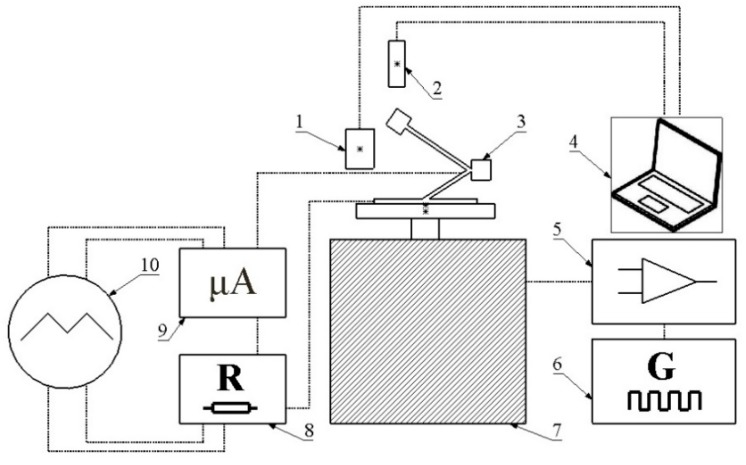
Experimental setup; 1,2—inductive displacement sensors; 3—prototype of the energy harvester; 4—computer with data management software; 5—power amplifier; 6—function generator; 7—electromagnetic shaker; 8—programmable resistance load; 9—micro current probe; 10—multichannel oscilloscope.

The experimental investigation was divided into the three steps i.e., resonant frequencies measurement, measurement of electrical output characteristics when harvester is excited by harmonic force at resonant frequencies and measurement of electrical output characteristics while energy harvester is excited by impact force. Experimental setup was built to perform all experimental investigations. It included two inductive displacement sensors (Models U3B and U20B, Lion precision, Oakdale, CA, USA), power amplifier (Type 2307, Brüel & Kjær, Nærum, Denmark), function generator (Model WW5064, Tabor Electronics, Tel Hanan, Israel), electromagnetic shaker (Model 4806, HP, Palo Alto, CA, USA), programmable custom made resistance load, micro current probe (µCurrent™, EEV blog, Sydney, Australia), multichannel oscilloscope (Model DLM2021, Yokogawa, Musashino, Tokyo, Japan), computer with data management software. Schematic of the experimental setup is given in Figure 14. Inductive sensors were used to control displacement of harvester’s base and tip. Function generator and amplifier were used to generate harmonic signal to control the displacement of the shaker. Programmable resistance load was used to apply different loads for the harvester. Moreover, multi channel oscilloscope was used to measure and to record current and voltage generated by the energy harvester.

**Figure 15:**
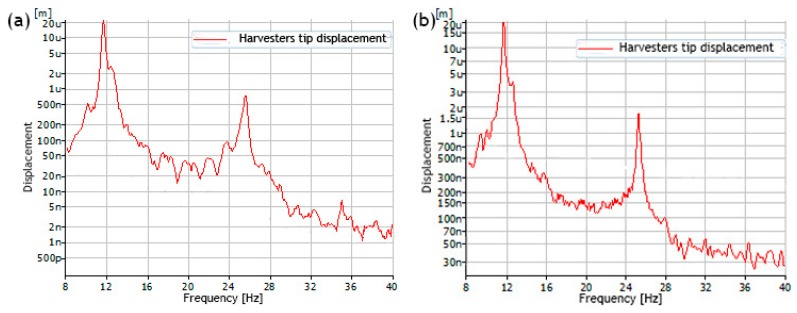
Frequency response characteristics of the bidirectional harvester when excitation direction align with *y* axis (**a**) and *z* axis (**b**).

Firstly, the frequency response characteristics were measured when excitation directions of the harvester were aligned to *y* and *z* directions. Inductive displacement sensors and computer with data acquisition software were employed for the measurement. Frequency range was set the same as used for numerical investigation i.e., from 8 Hz to 40 Hz. Acceleration amplitude of the shaker was set to 0.001 m/s^2^. Acceleration amplitude was reduced compare to the amplitude used during numerical simulation because of limitation of inductive displacement sensor. According to technical specification, sensor can measure displacement up to 0.5 mm while results of numerical investigation showed that tip displacement is much larger. Results of the measurement are shown in Figure 15.

It can be seen that during excitation in *y* and *z* directions, resonance frequencies occur at 11.25 Hz and 26.65 Hz. Resonance frequencies for both excitation cases are the same. Therefore, it can be concluded that bidirectional energy harvester has predictable characteristics during excitation in different directions. Moreover, comparison between results of numerical and experimental investigations showed that differences between resonance frequencies does not exceed 2.55 Hz for the first resonance and 0.75 Hz for the second resonance. Vibration amplitudes obtained during numerical and experimental investigations do not match because of different acceleration of the base. On the other hand, further investigations will be made with acceleration amplitude used in numerical investigations i.e., 0.1 m/s^2^

**Figure 16:**
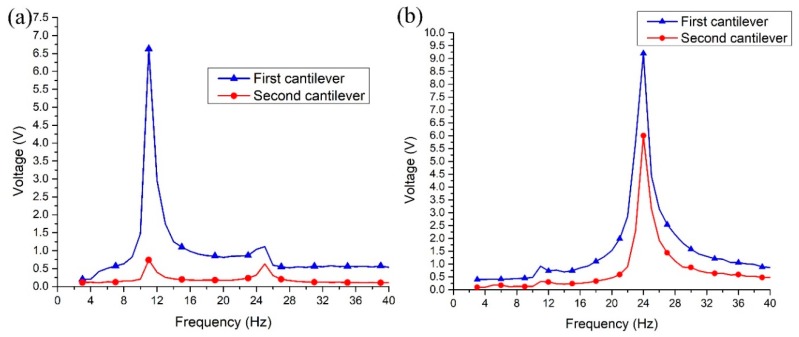
Measured open circuit voltage versus frequency when excitation direction is aligned with *y* axis (**a**) and *z* axis (**b**).

During the next experiment, open circuit voltage was measured in the frequency domain. Voltage was measured for each cantilever individually when excitation direction was aligned to *y* and *z* axis. Such way of the measurement was done to prevent charge cancelation because of the phase difference of output voltage. Results of measurements are shown in Figure 16.

Analyzing results shown in Figure 16a, it can be noted that the largest output voltage is generated at the first resonant frequency when excitation direction is aligned to *y* direction. The first cantilever generates the maximum voltage of 6.7 V, while the second cantilever generates 0.73 V. Therefore, energy harvester provides a total voltage of 7.43 V in parallel circuit case at the first resonance. The first cantilever generates the maximum voltage of 1.11 V and the second cantilever generates 0.63 V at the second resonant frequency. The total voltage of 1.73 V is obtained in parallel circuit case at the second resonant frequency.

Analysis of measurement results when excitation direction is aligned to *z* axis (Figure 16b) shows, that the maximum voltage of 9.2 V is obtained at the first cantilever and 6.1 V at the second cantilever when harvester vibrates at the second resonant frequency. Total voltage of 15.3 V is obtained in parallel circuit case. This value is 55.9% higher compare to the total voltage obtained when excitation direction was aligned to *y* axis. Moreover, the maximum output voltage generated by the first cantilever is 0.77 V while the second cantilever generates up to 0.24 V. In total the energy harvester generates 1.01V at the first resonant frequency during excitation in *z* direction.

Based on the results of the open circuit voltage measurement, it can be concluded that energy harvester can harvest kinetic energy when bidirectional excitation is applied. The first resonant frequency should be used as operating frequency when excitation direction is aligned to *y* axis and the second resonant frequency when excitation direction is aligned to *z* axis.

**Figure 17:**
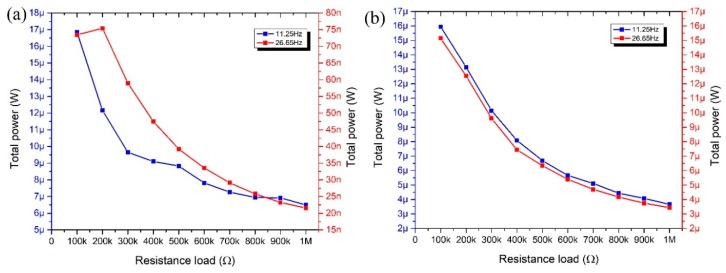
Measured electrical output power of bidirectional energy harvester when excitation direction is aligned with *y* axis (**a**) and *z* axis (**b**).

Electrical output power at the resonant frequencies was measured as well. Measurements were performed when different resistance loads were applied. The resistance range was from 100 kΩ to 1 MΩ. Results of measurements are presented in Figure 17.

Analyzing results shown in Figure 17a, it can be noted that the maximum output power of 16.85 µW was obtained at the first resonance when 100 kΩ resistance load was applied. The highest electrical power value of 73.45 nW was obtained with 200 kΩ resistance load when harvester vibrates at the second resonant frequency. Also, it can be seen that electrical power decreases when the resistance load is increasing at both resonant frequencies.

The energy harvester produces almost the same output power characteristics at the first and second resonances when excitation direction of the harvester is aligned to *z* axis. The highest output power was obtained at the same resistance load of 100 kΩ and is equal to 15.94 µW and 15.14 µW when energy harvester vibrates at the first and the second resonant frequency, respectively. Therefore, it can be concluded that energy harvester has better output power characteristics when excitation in the *z* direction is applied. Moreover, energy harvester can provide much higher output power at the second resonant frequency when excitation in the *z* direction is used.

Moreover, it can be concluded that optimal resistance load is 100 kΩ for the first resonant frequency and 200 kΩ for the second resonant frequency when excitation in the *y* direction is applied. Optimal resistance load for the energy harvester excited in the *z* direction is 100 kΩ for both resonant frequencies.

**Figure 18:**
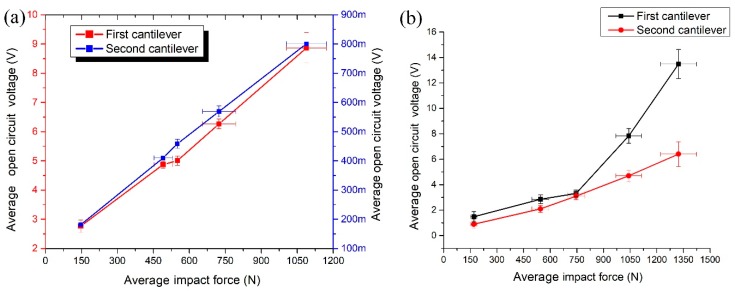
Measured open circuit voltage versus impact force when force direction is aligned with *y* axis (**a**) and *z* axis (**b**).

**Figure 19:**
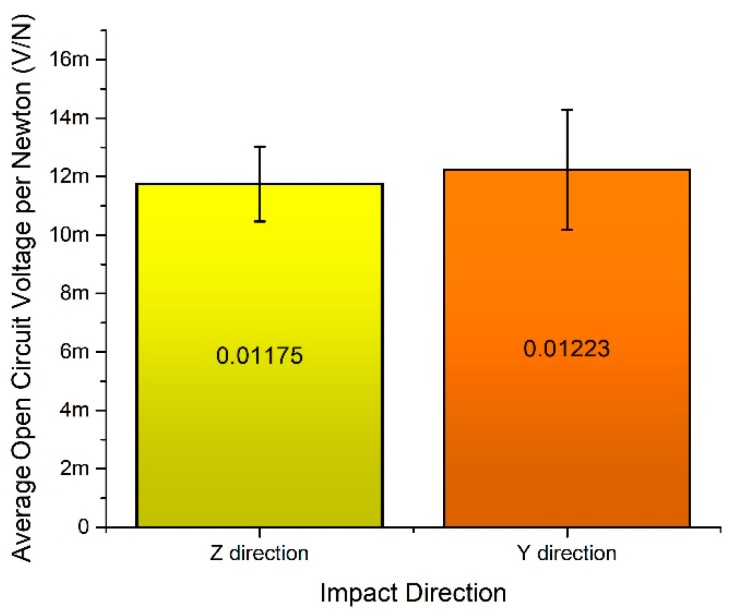
An average open circuit voltage per 1 N.

**Figure 20:**
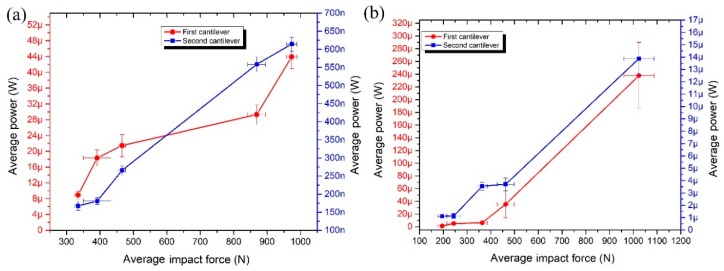
Measured average power versus impact force when force direction is aligned with *y* axis (**a**) and *z* axis (**b**).

**Figure 21:**
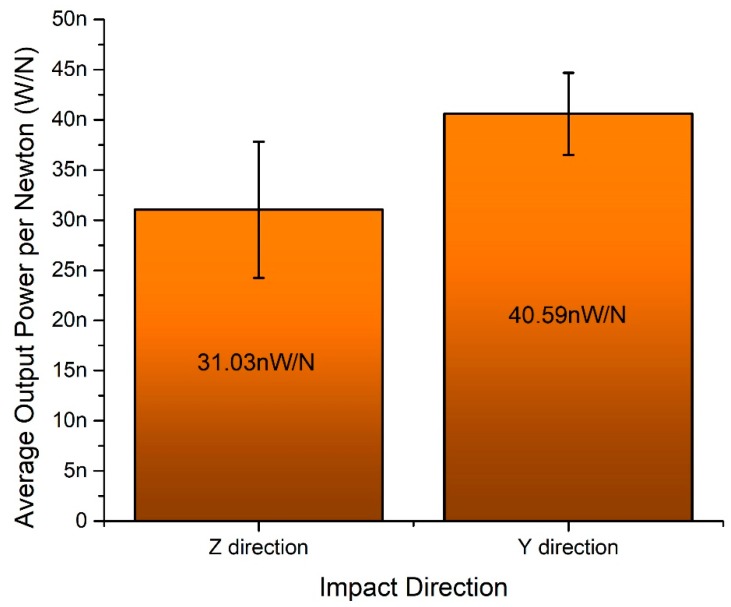
Characteristic of average output power per 1 N.

Next measurements were performed when an impact force is applied to the base of the harvester in *y* and *z* directions. Experimental setup with impact force sensor (Brüel & Kjær, Denmark) was used for this purpose. Dependence of open circuit voltage and electric output power from impact force was investigated. Results are shown in Figure 18, Figure 19, Figure 20 and Figure 21.

Measurements of open circuit voltage were performed from each cantilever individually as in the previous measurements. It can be seen that the first cantilever generates more than 11 times higher voltage compared to the voltage produced by the second cantilever when impact force direction is aligned to *y* axis (Figure 18a). The maximum voltage of 8.86 V is obtained from the first cantilever and 0.8 V from the second cantilever when the impact force of 1088.11 N is applied. Total voltage of 9.66 V is generated in parallel circuit case. The highest open circuit voltage of 13.48 V and 6.4 V was generated by the first and the second cantilever, respectively when impact force of 1322.3 N was applied to the *z* direction (Figure 18b). Total voltage is 19.88 V. Also, it can be seen that open circuit voltage increases almost linearly when the impact force increasing.

Average open circuit voltage per 1 N was calculated when the impact force is applied to *y* and *z* impact directions (Figure 19). It can be seen that energy harvester generates almost the same normalized average open circuit voltage value of 0.0117 mV/N and 0.0122 mV/N in *y* and *z* direction. Therefore, it can be concluded that energy harvester can provide stable electrical characteristics during impact based excitation in different directions.

Electric output power was measured as well when the impact force is applied in *y* and *z* directions (Figure 20). The highest average output power was generated by the first cantilever in both excitation cases. The electric output power of 43.93 µW and 61.42 nW was obtained from the first and the second cantilever respectively when the impact force of 973.5N was applied in *y* direction. The total maximum electric output power was 44.5 µW. The electric output power of 237.72 µW and 13.87 µW was obtained from the first and the second cantilever respectively when the impact force of 1023.38 N was applied in *z* direction. The total maximum electric output power was 251.59 µW. It must be noted that the second cantilever generates more than 20 times higher power compared to power generated when the impact force is aligned to the *y* axis.

Average electrical output power per 1 N was calculated when the impact force is applied to *y* and *z* impact directions (Figure 19). It can be seen that energy harvester generates 31.2 nW/N and 40.5 nW/N normalized electric power when the impact force is applied in *y* and *z* direction, respectively. The difference is 22.96%.

## 5. Conclusions

Numerical and experimental investigations showed that the proposed design of bidirectional piezoelectric energy harvester is suitable for application when areas are filled by kinetic energy which acts in several directions. Compare to ordinary cantilever based energy harvesting systems the proposed design of energy harvester is more suitable for real-life applications. The ability to capture kinetic energy with varying force vectors makes the system more flexible for areas with complex applications problems and scopes. The bidirectional energy harvester can operate with high acceleration amplitudes and provide stable power output during harmonic excitation i.e., 16.85 µW and 15.94 µW in *y* and *z* directions, respectively. Such power level can be achieved during excitation systems operation in continuous mode. On the other hand, the energy harvester can provide 3.1 nW/N and 4.05 nW/N output power characteristics in *y* and *z*-direction during impact based excitation. In summary, it can be concluded that the proposed design of bidirectional energy harvester eliminates demand to exact excitation force alignment to the energy harvester and provides the possibility to solve more complex energy harvesting problems in real-life applications.

## Figures and Tables

**Table 1:** Material properties.

Material Properties	Aluminum Alloy 6061	Piezo Ceramic PIC255
Density [kg/m^3^]	2700	7800
Young’s modulus [N/m^2^]	6.89 × 10^10^	-
Poisson’s ratio	0.33	-
Isotropic structural loss factor	0.02	0.015
Relative permittivity	-	In the polarization direction ε_33_^T^/ε_0_ = 1200; Perpendicular to polarity ε_11_^T^/ε_0_ = 1500
Elastic stiffness coefficient c_33_^D^, [N/m^2^]	-	16.6 × 10^10^
Dielectric loss factor—tan δ [10^−3^]	-	20
Coupling factor k_31_	-	0.35
Piezoelectric voltage coefficient g_31_ [10^−3^ Vm/N]	-	–11.3

**Table 2:** The initial values of the parameters used for optimization.

Parameter	Value
L_min_	75 mm
M_min_	3 g
α_min_	1.13 rad

**Table 3:** Geometrical and physical parameters of the harvester.

Parameter	Value	Description
L_1_	83 mm	Length of first cantilever
L_2_	79 mm	Length of second cantilever
t	0.6 mm	Thickness of the cantilevers
PZT_1_	0.9 L_1_, mm	Length of piezo ceramics placed on L_1_
PZT_2_	0.9 L_2_, mm	Length of piezo ceramics placed on L_2_
t_pzt_	0.2 mm	Thickness of piezo ceramic layer
t_total_	1 mm	Thickness of bimorphs
α_1_	2.75 rad°	Angle between clamping base and L_1_
α_2_	1.38 rad°	Angle between L_1_ and L_2_
M_1_	3.04 g	Seismic mass placed at junction between L_1_ and L_2_
M_2_	4.25 g	Seismic mass placed at tip of L_1_
w	5 mm	Width of whole energy harvester
